# Molecular Cloning and Expression Analysis of Lactate Dehydrogenase from the Oriental River Prawn *Macrobrachium nipponense* in Response to Hypoxia

**DOI:** 10.3390/ijms19071990

**Published:** 2018-07-08

**Authors:** Shengming Sun, Hongtuo Fu, Jian Zhu, Xianping Ge, Xugan Wu, Hui Qiao, Shubo Jin, Wenyi Zhang

**Affiliations:** 1 Key Laboratory of Genetic Breeding and Aquaculture Biology of Freshwater Fishes, Ministry of Agriculture, Freshwater Fisheries Research Center, Chinese Academy of Fishery Sciences, Wuxi 214081, China; sunsm@ffrc.cn (S.S.); gexp@ffrc.cn (X.G.); qiaoh@ffrc.cn (H.Q.); jinsb@ffrc.cn (S.J.); zhangwy@ffrc.cn (W.Z.); 2 Key Laboratory of Exploration and Utilization of Aquatic Genetic Resources, Shanghai Ocean University, Ministry of Education, Shanghai 201306, China; xgwu@shou.edu.cn

**Keywords:** hypoxia, lactate dehydrogenase, hypoxia inducible factor, promoter, *Macrobrachium nipponense*

## Abstract

Metabolic adaption to hypoxic stress in crustaceans implies a shift from aerobic to anaerobic metabolism. Lactate dehydrogenase (LDH) is a key enzyme in glycolysis in prawns. However, very little is known about the role of LDH in hypoxia inducible factor (HIF) pathways of prawns. In this study, full-length cDNA of LDH (MnLDH) was obtained from the oriental river prawn *Macrobrachium nipponense*, and was characterized. The full-length cDNA is 2267-bp with an open reading frame of 999 bp coding for a protein of 333 amino acids with conserved domains important for function and regulation. Phylogenetic analysis showed that MnLDH is close to LDHs from other invertebrates. Quantitative real-time PCR revealed that MnLDH is expressed in various tissues with the highest expression level in muscle. MnLDH mRNA transcript and protein abundance in muscle, but not in hepatopancreas, were induced by hypoxia. Silencing of hypoxia-inducible factor 1 (HIF-1) α or HIF-1β subunits blocked the hypoxia-dependent increase of LDH expression and enzyme activity in muscle. A series of MnLDH promoter sequences, especially the full-length promoter, generated an increase in luciferase expression relative to promoterless vector; furthermore, the expression of luciferase was induced by hypoxia. These results demonstrate that MnLDH is probably involved a HIF-1-dependent pathway during hypoxia in the highly active metabolism of muscle.

## 1. Introduction

The higher lactate levels of hemolymph indicate that anaerobic metabolism makes a significant contribution to energy production in crustaceans during hypoxia [1]. Lactate dehydrogenase (LDH, EC 1.1.1.27) as an enzyme is found in almost all organisms, LDH catalyzes the conversion of lactate to pyruvic acid and back, as it converts NAD^+^ to NADH and back [2]. In mammals, different LDH subunits (isoforms) can be included in LDH tetramers, such as LDH-A (mainly present in anaerobic muscle tissue), LDH-B (mainly present in aerobic heart tissue), and LDH-C (present only in mature testis), which show different tissue expression, kinetic, physicochemical and immunochemical properties [3]. However, there is much less information on LDH in invertebrates. In fact, most invertebrates preferentially oxidize L-lactic acid, several species of mollusks, a few arthropods and polychaetes were found to have exclusively D-LDH enzymatic activity [4,5], and all LDHs from crustaceans have tetrameric structure [6,7,8] with around 140 kDa, 36 kDa per subunit [9]. Various invertebrates, including northern krill *Meganyctiphanes norvegica* [10,11], the Antarctic krill *Euphausia superba* [12] and the snow crab *Chionoecetes opilio* [13] contain multiple forms of LDH.

*Macrobrachium nipponense*, the oriental river prawn, is an economically important aquaculture animal with an annual production value of almost 20 billion RMB. Prawns are relatively susceptible to hypoxia compared with most crustaceans [14]. Thus, in prawn production, hypoxia may cause large economic losses because of increased mortality and decreased growth rate. Our previous studies have focused on energy metabolic mechanism for prawns in response to hypoxia stress [15,16]. However, little information is available on expression and regulation of LDH, a key enzyme of anaerobic metabolism, in *M. nipponense*. Understanding the glycolytic flux induced by hypoxia in *M. nipponense* is essential for successful high-density prawn aquaculture.

In mammals, LDH mRNA expression is directly upregulated by hypoxia-inducible factor 1 (HIF-1) [17,18]. We hypothesized that in hypoxic *M. nipponense*, LDH (MnLDH) is similarly regulated by HIF-1. Here, we obtained and characterized the full-length MnLDH cDNA. We compared the expression levels and activity of MnLDH in response to hypoxia after silencing HIF-1 by RNA interference (RNAi). The promoter region of the *M. nipponense* LDH gene was also analyzed and its activity assessed in the Drosophila S2 cell culture system.

## 2. Results and Discussion

### 2.1. Characteristics and Phylogeny of MnLDH

**Figure 1:**
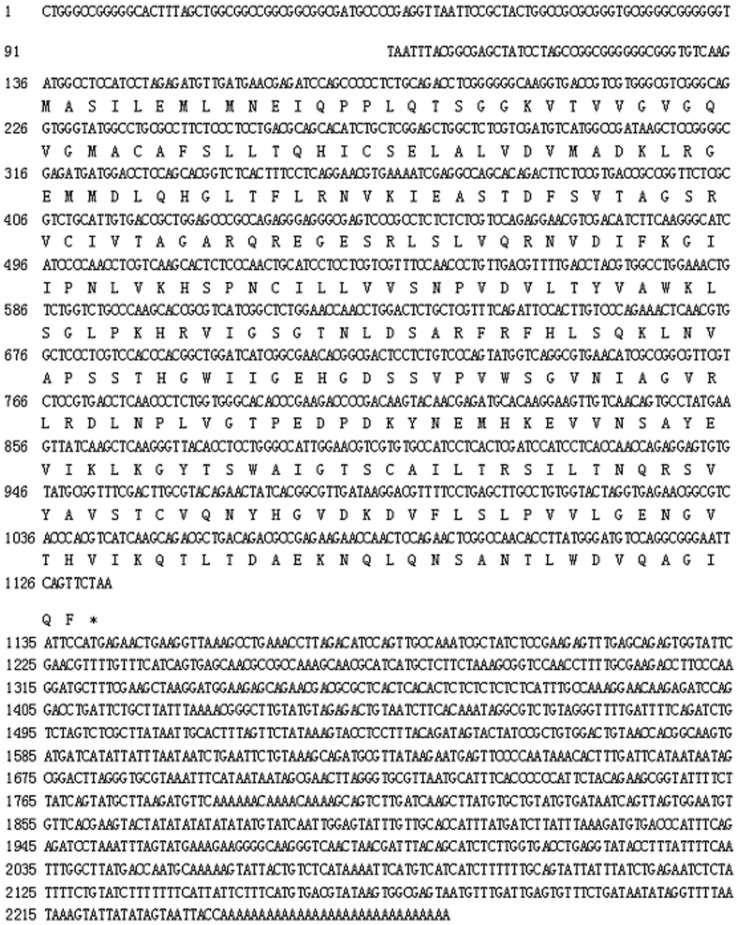
Nucleotide and predicted protein sequences of *Macrobrachium nipponense* LDH. The asterisk indicates a stop codon.

**Figure 2:**
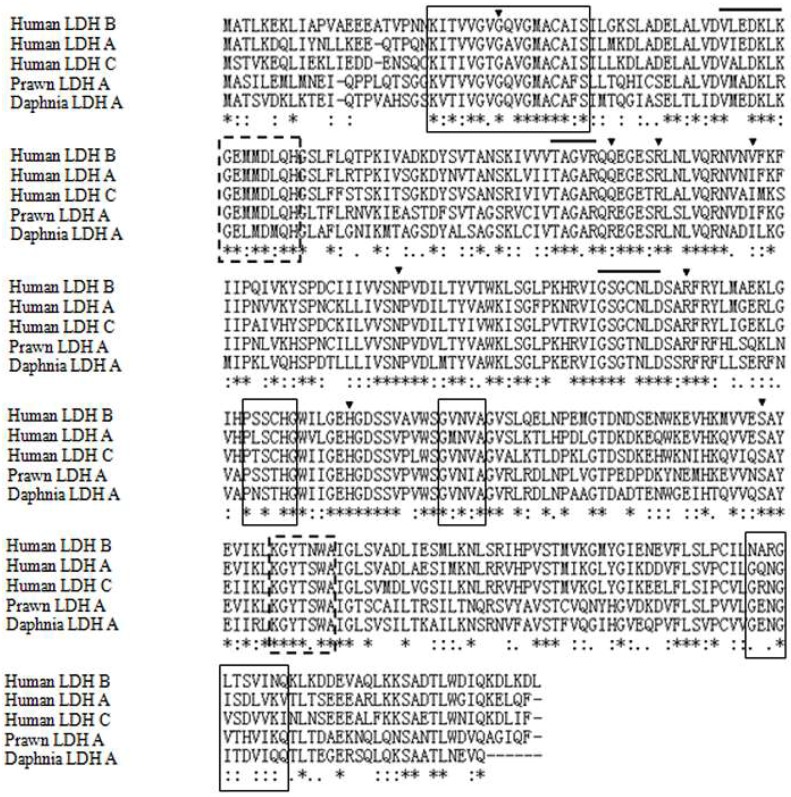
Multiple sequence alignment of LDH amino acid sequences. The LDHs of *Macrobrachium nipponense* (Prawn LDH), *Daphnia pulex* (Daphnia LDH-A) and *Homo sapiens* (Human LDH-A, -B and -C) sequences are included in the analysis. (*) identical residues; (:) conservative substitutions; and (.) semi-conservative substitutions. The residues involved in the binding sites for substrate and NAD^+^, and the dimer and tetramer interfaces, are marked in triangle and with a line above, a dashed box and a black solid box, respectively.

**Figure 3:**
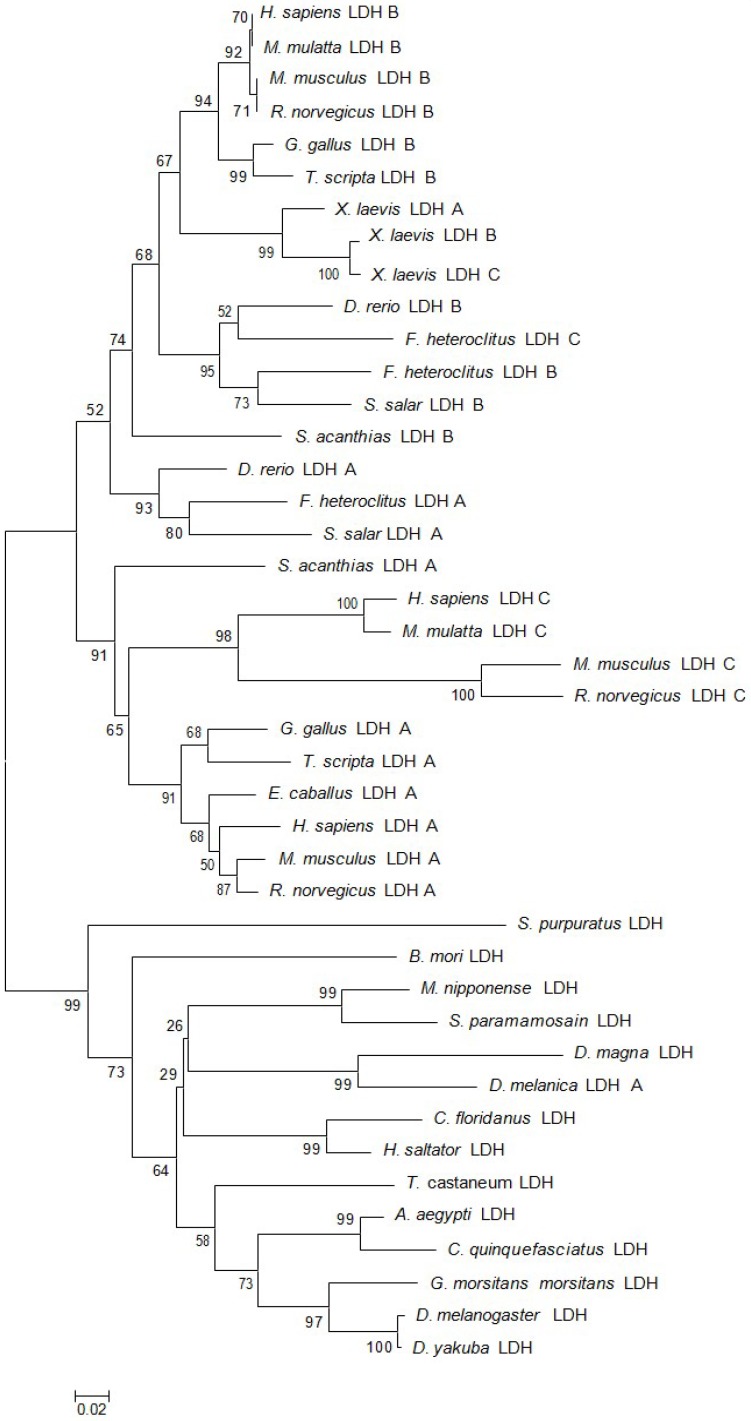
Phylogenetic trees were generated using the neighbor-joining method based on the alignment of amino acid sequences of LDH. The numbers shown at the branches indicate the bootstrap values (%). Sequences used in the analysis with their abbreviation and GenBank accession number are: *Macrobrachium nipponense*, MF033360 (this work); *Homo sapiens* LDH A, AAH67223; *H. sapiens* B, AAH71860; *H. sapiens* C, AAH64388; *Macaca mulatta* (monkey) B, XP_001085541; *M. mulatta* C, XP_001082436; *Mus musculus* (mouse) A, NP_034829; *M. musculus* B, NP_032518; *M. musculus* C, NP_038608; *Rattus norvegicus* (rat) A, NP_058721; *R. norvegicus* B, NP_058962; *R. norvegicus* C, NP_036727; *Gallus gallus* (chicken) A, NP_990615; *G. gallus* B, AAG48560; *Trachemys scripta* (pond slider) A, AAD46979; *T. scripta* B, AAD46980; *Xenopus laevis* (frog) A, NP_001081050; *X. laevis* B, NP_001080852; *X. laevis* C, NP_001165451; *Danio rerio* (zebrafish) A, NP_571321; *D. rerio* B, NP_571322; *Fundulus heteroclitus* (mummichog) A, Q92055; *F. heteroclitus* B, AAA49305; *F. heteroclitus* C, Q06176; *Salmo salar* (Atlantic salmon) A, NP_001133114; *S. salar* B, ACI34235; *Squalus acanthias* (spiny dogfish) A, AAA91038; *S. acanthias* B, AAD02703; *Equus caballus* (horse) A, ADG85262; *Strongylocentrotus purpuratus* (purple urchin) LDH, XP_791548; *Bombyx mori* (silkmoth) LDH, ABS18410; *Scylla paramamosain* (crab), ACY66479; *Daphnia magna* (water flea), ACN51907; *Daphnia melanica* (water flea) A, AEK84351; *Camponotus floridanus* (ant), EFN62194; *Harpegnathos saltator* (ant), EFN76243; *Tribolium castaneum* (red flour beetle), XP_968203; *Aedes aegypti* (mosquito), XP_001662150; *Culex quinquefasciatus* (mosquito), XP_001866924; *Glossina morsitans morsitans* (tsetse fly), ADD18974; *Drosophila melanogaster* (fruit fly), AAB07594; *Drosophila yacuba* (fruit fly), XP_002093920.

The full-length MnLDH cDNA fragment was 2267 bp (GenBank Accession No. MF033360), including an open reading frame of 999 bp (Figure 1). The molecular mass of the putative protein was 36.16 kDa, with pI 7.14. Comparison of the predicted MnLDH protein sequence with LDHs from other species showed conserved binding sites for substrate and NAD^+^, as well as dimer and tetramer interfaces (Figure 2). Amino acid sequence comparison showed that highly conserved residues were found in all LDHs, for example for substrate binding, and dimer/tetramer interface formation. MnLDH has high identity to LDHs from crustaceans such as the crab *Scylla paramamosain* and the water fleas *Daphnia magna* and *D. melanica*. Interestingly, similar to previous study in the shrimp *Litopenaeus vannamei* [19], we found that MnLDH contains active site residues found in vertebrate LDH subunits. For example, Gln30 and Ala98 are found in the active site of vertebrate LDH B, and Ile116 in vertebrate A and C subunits [20]. In phylogenetic analysis based on amino acid sequences, MnLDH occupied a separate branch at the base of invertebrate LDHs (Figure 3).

### 2.2. Tissue-Specific Expression of MnLDH

**Figure 4:**
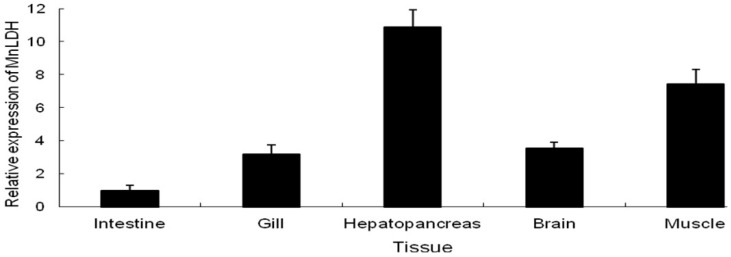
Quantitative real-time PCR analysis of LDH mRNA expression in various tissues of *M. nipponense*. The ratio compares the gene expression in different tissues to that in heart. The β-actin gene was used as an internal control. Vertical bars represent mean ± standard error of the mean (SEM) values for triplicate samples.

qRT-PCR was used to examine the expression pattern of MnLDH in different tissues of *M. nipponense*, including hepatopancreas, intestine, heart, muscle, gill. MnLDH mRNA was found to be constitutively expressed in all examined tissues. MnLDH transcripts were present at the highest level in muscle, and at a lower level in the intestine (Figure 4). Some invertebrates may possess single form of LDH enzyme and mainly present in muscle tissue, such as crayfish *Orconectes limosus* [21], shrimp *Palaemon serratus* [22], and common shrimp *Crangon crangon* [7], which were similar with present study where we observed the highest mRNA expression of MnLDH in muscle tissue. Considering that muscle is an anaerobic tissue that functions in locomotion and gluconeogenesis, suggesting that MnLDH is principally expressed in anaerobic tissues and involved in metabolism by glycolysis. In contrast, the intestine is organ involving in digest food and absorbs nutrients in invertebrates, which were mainly related to with metabolic changes by variation of intestinal microflora, thus we observed the lowest MnLDH mRNA expression in intestine.

### 2.3 Expression and Purification of Recombinant MnLDH

**Figure 5:**
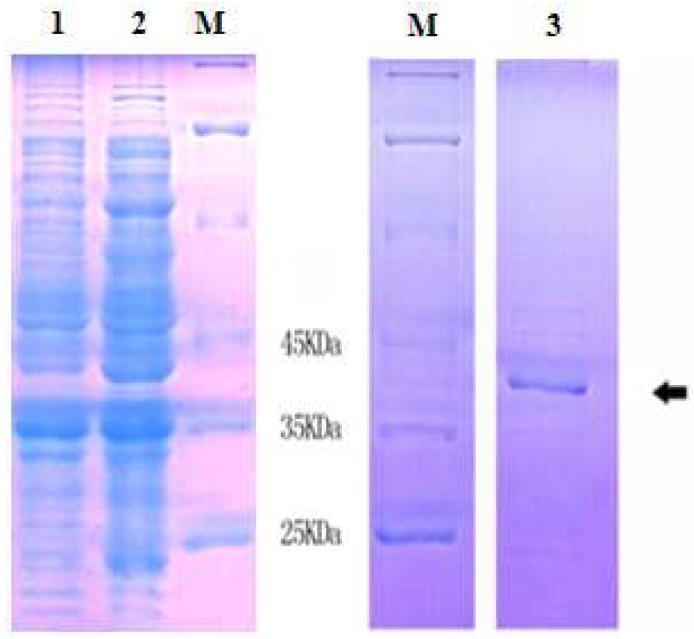
SDS-PAGE analysis of *M. nipponense* LDH protein purification. M, molecular mass standards; lane 1, *Escherichia coli* crude extract, without induction; lane 2, induced expression of recombinant MnLDH for 2 h; lane 3, purified protein as shown in black arrow.

The 999-bp DNA encoding MnLDH was cloned into expression vector pET28a and expressed in *Escherichia coli*. By SDS-PAGE, a ~41 kDa band of recombinant protein was observed (Figure 5), in agreement with the theoretical molecular weight of the LDH subunit of ~36 kDa plus His tag derived from the vector. Figure 5B (lane 3) shows SDS-PAGE of purified recombinant MnLDH. The molecular weight of rMnLDH was similar to other animal’s LDH [23,24,25].

### 2.4. MnLDH Expression in Muscles and Hepatopancreas of Prawns during Hypoxia

**Figure 6:**
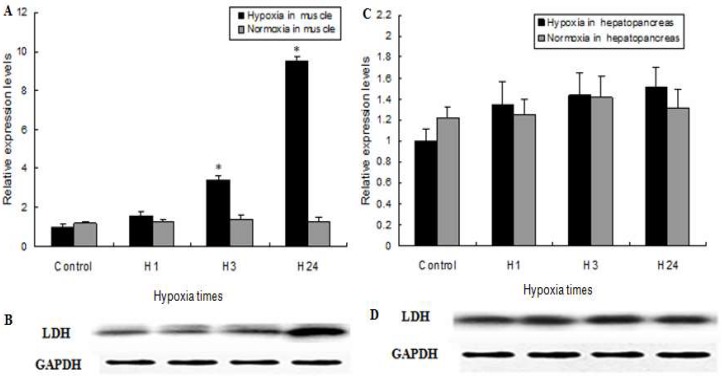
LDH mRNA and protein abundance in muscle (**A**,**B**) and hepatopancreas (**C**,**D**) from *M. nipponense* exposed to hypoxia. (*) indicates significant differences (*p* < 0.05) between the normoxia and hypoxia groups. Vertical bars represent mean ± SEM values for triplicate samples.

Lactate dehydrogenase catalyzes the interconversion of pyruvate and lactate in mammalian cells this is regulated by the oxygen level [26]. The prawn *M. nipponense* is cultured in ponds that frequently suffer hypoxia, especially when water temperatures of pond were more than 30 °C in summer [27]. It is therefore important to understand the molecular response and adaptation of these prawns to hypoxia. MnLDH mRNA significantly increased in muscle of prawns subjected to hypoxia for 3 h compared to prawns treated by normoxia, and it reached the highest level detected after 24 h of hypoxia. No significant changes of MnLDH transcripts were observed in response to hypoxia for 1 h (Figure 6A,B). In the present study, the amount of LDH protein in muscle of *M. nipponense* did not change after 1 h of hypoxia but significantly increased after 24 h of hypoxia. Notably, however, in hepatopancreas, there were no significant differences in MnLDH mRNA expression or LDH protein abundance in response to hypoxia (Figure 6C,D), suggesting there is tissue-specific regulation of LDH in prawns. Previous studies have investigated the expression patterns of carbohydrate metabolism enzymes genes in different tissues of shrimp under hypoxia, such as hexokinase, phosphofructokinase, fructose 1,6-bisphosphatase and phosphofructokinase [28,29,30]. Lactate, an end-product of glycolysis, is transported by the blood to the liver and reconverted to glucose and glycogen by LDH to meet energy needs under hypoxic stress. Thus, elevation of LDH activity in muscle may enhance anaerobic metabolism to maintain energy for survival. The observed tissue-specific expression of LDH indicates that *M. nipponense* shifts respiratory metabolism to anaerobiosis when oxygen availability is low.

### 2.5. LDH mRNA Levels and Enzyme Activity Are Affected by HIF-1 Silencing

**Figure 7:**
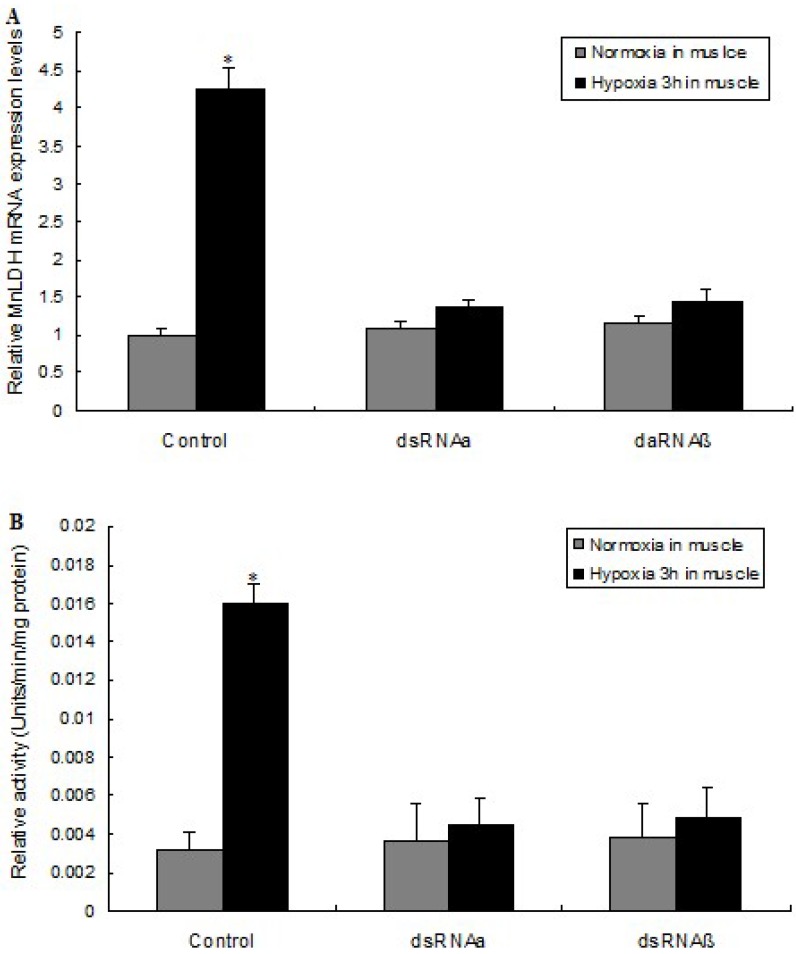
LDH mRNA level (**A**) and enzyme activity (**B**) in muscle from *M. nipponense* exposed to hypoxia and HIF-1 silencing. The prawns were exposed to hypoxia (2.0 mg/L O_2_ for 1 h) and injected with saline solution (control), dsRNAα or dsRNAβ. * indicates significant differences (*p* < 0.05) between the control and dsRNAα groups, or between the control and dsRNAβ, respectively. Vertical bars represent mean ± SEM values for triplicate samples.

In the control group (no RNAi), after 3 h of hypoxia, the MnLDH mRNA level in muscle increased 4.2-fold compared to normoxia. In contrast, no significant changes in response to 3-h hypoxia were observed in the level of the MnLDH transcript in prawns where HIF-1 α or β were silenced by RNAi (Figure 7A). In unsilenced prawns, LDH enzyme activity was 5.1-fold higher after 3-h hypoxia than in normoxic prawns. However, when HIF-1 was silenced, hypoxia did not result in a change in LDH activity (Figure 7B). We previously showed a hypoxia-induced increase in the expression of HIF-1α and HIF-1β in M. nipponense [31], which suggested that HIF-1 participates in the response to hypoxia in this organism. Here, silencing of the HIF-1 α or β subunits in muscle blocked the induction of LDH expression. Further, we analysis MnLDH promotor sequences in order to understand the transcriptional regulation of MnLDH by HIF-1 in prawns responsed to hypoxia.

### 2.6. Analysis of the MnLDH promotor

**Figure 8:**
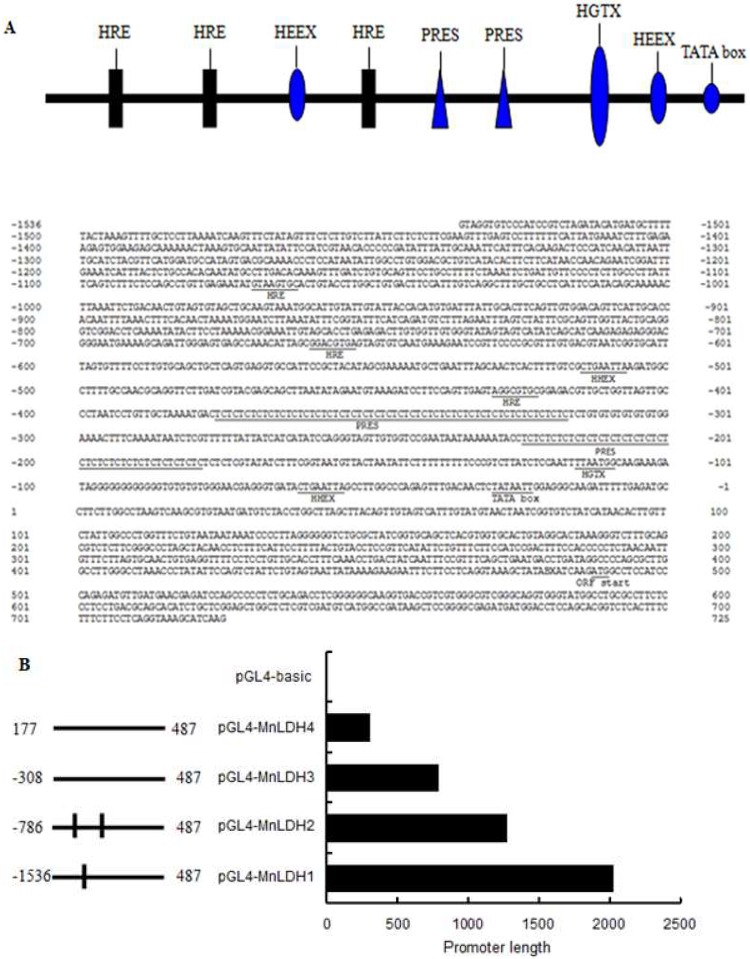

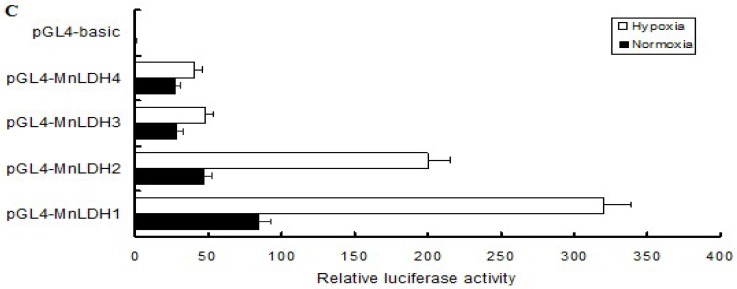
The 5′-flanking sequence of the *LDH* gene of *M. nipponense*. Schematic diagram of the gene promoter region (**A**). Putative transcription factor binding sites are underlined and labeled. Putative elements including HIF-1a binding sites (HRE; black bars), DNA detection reaction elements (PRES; blue triangle), a Nkx6B-like homeodomain protein binding site (HGTX; blue large oval), transcriptional inhibitory factor binding sites (HEEX; blue medium-sized oval) and a TATA box (blue small oval) are shown. (**B**) Expression of MnLDH promoter constructs in transiently-transfected insect Sf9 cells. A schematic illustration of the promoter-luciferase reporter constructs is shown on the left, HREs are indicated by I. (**C**) The activity of each construct relative to empty vector (pGL3-basic) in transiently-transfected Sf9. The data are expressed as fold-induction relative to empty vector (pGL3-basic), and the error bars represent ± SE of three replicate trials.

The 5′-flanking sequence (2017 bp) of the *MnLDH* gene in muscle was identified by genome walking. It contains a TATA box (TATAA, 50 bp before the ATG start codon), but no CAAT or GC box. Many hypoxia response elements (HSEs) were predicted in the upstream sequence of MnLDH (Figure 8A) using the TRANSFAC database; they may be involved in transcriptional regulation of the gene. A series of MnLDH promoter fragments were cloned upstream of a luciferase reporter gene and the constructs were transfected into *Drosophila* S2 cells (Figure 8B). Relative to empty vector (pGL3-Basic), the full-length MnLDH promoter (pGL3-MnLDH1) resulted in an 85-fold increase in luciferase activity in normoxia condition. Deletion of two HSEs (pGL4-MnLDH2) decreased the activity by about half. Deletion of three HSEs (pGL4-MnLDH3, pGL4-MnLDH4) significantly decreased (by about 80%) the promoter activity relative to the full-length promoter. Hypoxia increased MnLDH1 promoter activity significantly relative to normoxia (Figure 8C). HIF-1 may recognize HREs containing a “CACGTG” sequence present in the LDH promoter region. In mammals cells, the genes encoding several glycolytic and gluconeogenic enzymes are transcriptionally regulated by hypoxia [32,33,34,35]. HIF-1 appears to increase transcription of LDH A via HREs in response to hypoxia [36], which was consistent with the present study where many HREs were predicted in the upstream sequence of MnLDH, and the deletion of HREs decreased the transcriptional activity of the MnLDH promotor. Deletion of more HREs further reduced gene expression. These results suggest that activated HIF-1 binds to the HREs and initiates transcription, consistent with previous reports in mammals [37,38,39,40,41].

## 3. Materials and Methods

### 3.1. Experimental Animals and Hypoxia Treatment

Healthy prawns (*n* = 300; 1.88–2.78 g) were transferred from a prawn breeding base near Tai Lake in Wuxi, China (120°13′44” E, 31°28′22” N) to the laboratory and allowed to adapt to the new environment for 2 weeks. Two hundred and forty prawns were randomly divided into two groups for hypoxia challenge with triplicates for each group (i.e., 40 prawns per replicate). Control prawns were maintained in normoxic conditions (6.5 ± 0.2 mg O_2_ L^−1^) and hypoxic conditions were 2.0 ± 0.2 mg O_2_ L^−1^ as described previously [42], which were maintained by bubbling with N_2_ gas until the desired O_2_ concentrations were reached; oxygen levels were maintained by adding N_2_ gas when needed. Samples including hepatopancreas, gills, muscle, intestine, and brain were harvested and stored at −80 °C. All animals were conducted in accordance with the “Guidelines for Experimental Animals” of the Chinese Academy of Fishery Sciences. The study protocols (FFRC125) were approved by the Institute for Experimental Animals of Chinese Academy of Fishery Sciences on 26 August 2016.

### 3.2. Cloning of Complete Sequence and Upstream Sequence of MnLDH

A 3′-full RACE Core Set Ver. 2.0 kit and a 5′-full RACE kit (TaKaRa, Dalian, China) were used to determine the cDNA 3′- and 5′-ends of the *MnLDH* gene. The primers used in this cloning are listed in Table 1. PCR products were purified using a gel extraction kit (Sangon, Shanghai, China), followed by sequencing using an ABI3730 DNA analyzer. The 5ʹ- promoter region of *MnLDH* was cloned using a genome walking kit (TaKaRa, Dalian, China) with primers GSPR1, GSPR2, GSPR3 by previous methods [43]. A 2017-bp DNA fragment was obtained and cloned into vector pMD18-T (Takara, Dalian, China) for sequencing. Nucleotide sequence and bioinformatic analyses were performed as described previously [44].

### 3.3. Expression and Purification of MnLDH and Antibody Production

MnLDH cDNA was subcloned into vector pET-28a (Novagen, Darmstadt, Germany) between the BamHI and XhoI sites according to our previous method [45]. Overexpression of the recombinant protein, purification of recombinant MnLDH (rMnLDH), and production of rabbit LDH antibodies using rMnLDH were performed as our previously described [45].

### 3.4. Quantitative Real-Time (qRT)-PCR and Western Blotting Analysis of MnLDH Expression of Prawns in Response to Hypoxia

The expression level of MnLDH in different tissues and hypoxia treatment was detected by qRT-PCR as described previously [46]. Briefly, qRT-PCR was performed using a Prime Script RT reagent kit (TaKaRa) and a Bio-Rad iCycler iQ5 Real-Time PCR system (Bio-Rad, Hercules, CA, USA). Primers for PCR are listed in Table 1 and PCR conditions were as described [47]. Western blot analysis to detect LDH protein was performed as previously described [45]. Muscle and hepatopancreas sampled after 0, 1, 3, and 24 h of hypoxic treatment were homogenized and then centrifuged at 10,000× *g* for 20 min at 4 °C. Proteins (30 μg) were separated by 12% SDS-PAGE and transferred to nitrocellulose membranes. The membrane was incubated with rabbit anti-LDH antibody (1:1000) for 1 h at room temperature, followed by the addition of horseradish peroxidase-linked secondary antibody (1:2000) and diaminobenzidine for visualization of bands.

### 3.5. HIF-1 Silencing and Hypoxia

HIF-1 silencing and hypoxia were performed as described previously [48]. Briefly, dsRNA of MnHIF-1α (KP050352) and MnHIF-1β (KP050353) were synthesized, followed by intramuscular injection at 4 μg/g of body weight (α or β, separately) to prawns (*n* = 10) which were then subjected to normoxia for 24 h or hypoxia for 1, 3 or 24 h. Prawns injected with saline were used as controls. The muscle tissues were harvested and stored at −80 °C for gene expession and enzymatic activity assay.

### 3.6. Enzymatic Activity Assay

LDH activity was measured using a previously reported method [6]. Acetone powder was prepared from homogenized muscle (100 mg) in four volumes (*w*/*v*) of 0.1 M Tris-HCl, 5 mM-mercaptoethanol, pH 8, precipitated by adding eight volumes of acetone. LDH activity is reported in micromoles of NAD^+^ formed per min at 25 °C per mg of protein [49]. Sodium pyruvate was omitted as controls were run concurrently for LDH measurement.

### 3.7. Construction, Transfection and Activity Assay of Luciferase Plasmids

A series of truncated promoters of MnLDH were generated by PCR using primers P1, P2, P3 and P4 (Table 1) and subcloned into pGL4-Basic firefly luciferase reporter vector in the KpnI/XhoI sites, resulting in plasmids pGL4-LDH1, pGL3-LDH2, pGL3-LDH3, and pGL3-LDH4 respectively. The luciferase assay and transfection experiments in normoxia and hypoxia condition were performed as previously described [50], respectively.

### 3.8. Statistical Analysis

All experiments were performed at least in triplicate and data are presented as the mean ± SE. SPSS 19.0 software (SPSS Inc., Chicago, IL, USA) was used for all analyses. Student’s *t*-test was used to compare all pairs of means. *p* < 0.05 was considered statistically significant.

## 4. Conclusions

In summary, this study cloned and characterized the *LDH* gene of the oriental river prawn *M. nipponense*. Hypoxia increases MnLDH mRNA expression in a tissue-specific manner. hypoxia-induced LDH gene expression is regulated in a HIF-1 dependent manner, and that this cellular response is oriented to ensure the contribution of LDH in muscle to accelerate the rate of glycolysis in order to generate energy. Further study would clarify the energy metabolic mechanism in muscle tissue when prawns were exposed to hypoxia, which will help selective breeding of hypoxic tolerance prawn. 

## Figures and Tables

**Table 1:** Primers used in this study.

Primer	Primer Sequence (5′-3′)
MnLDH-F1 (5′-RACE out primer)	GTTCTCGCGTCTGCATTGTG
MnLDH-F2 (5′-RACE in primer)	TACGTGGCCTGGAAACTGTC
MnLDH-R1 (3′-RACE out primer)	TTGAGGTCACGGAGACGAAC
MnLDH-R2 (3′-RACE in primer)	TTGGTTCCAGAGCCGATGAC
MnLDH-F (real-time primer)	CTGTCCCAGTATGGTCAGGC
MnLDH-R (real-time primer)	CCGCATACACACTCCTCTGG
GSP-R1 (genome walking)	GGAAAGTGAGACCGTGCT
GSP-R2 (genome walking)	GAGCTTATCGGCCATGAC
GSP-R3 (genome walking)	GGGCTGGATCTCGTTCAT
P1-F (promoter activity) −1536~487	CATTTCTCTGGCCTAACTGGCCGGTACCGTAGGTGTCCCATCCGTCTAGATAC
P1-R (promoter activity) −1536~487	CGAGGCCAGATCTTGATATCCTCGAGCTTGATGCTTTACCTGAGGAAG
P2-F (promoter activity) −786~487	CATTTCTCTGGCCTAACTGGCCGGTACCTATACTTCCTAAAAACGGAAATTG
P2-R (promoter activity) −786~487	CGAGGCCAGATCTTGATATCCTCGAGCTTGATGCTTTACCTGAGGAAG
P3-F (promoter activity) −308~487	CATTTCTCTGGCCTAACTGGCCGGTACCGTGTGTGGAAAACTTTCAAAATAATC
P3-R (promoter activity) −308~487	CGAGGCCAGATCTTGATATCCTCGAGCTTGATGCTTTACCTGAGGAAG
P4-F (promoter activity) 177~487	CATTTCTCTGGCCTAACTGGCCGGTACCGCACTAAAGGGTCTTTGCAG
P4-R (promoter activity) 177~487	CGAGGCCAGATCTTGATATCCTCGAGCTTGATGCTTTACCTGAGGAAG
MnpLDH CDS amplification (BamHI)	CGGGATCCATGGCCTCCATCCTAGAGATG
MnpLDH CDS amplification (XhoI)	CCGCTCGAGGAACTGAATTCCCGCCTGGAC
β-Actin F (real-time primer)	TATGCACTTCCTCATGCCATC
β-Actin R (real-time primer)	AGGAGGCGGCAGTGGTCAT

## Abbreviations

LDH	Lactate dehydrogenase
qRT-PCR	Quantitative real-time reverse transcription PCR
RACE	Rapid amplification of cDNA ends
HIF	Hypoxia inducible factor
DO	Dissolved oxygen
HRE	Hypoxia response elements
SDS	Sodium dodecyl sulfate
IPTG	Isopropyl-β-d-galactopyranoside
dsRNA	Double-stranded RNA
ATP	Adenosine triphosphate
EDTA	Ethylenediaminetetraacetic acid
PAGE	polyacrylamide gel electrophoresis
